# A tRNA-derived RNA Fragment Plays an Important Role in the Mechanism of Arsenite -induced Cellular Responses

**DOI:** 10.1038/s41598-018-34899-2

**Published:** 2018-11-15

**Authors:** Shengxuan Liu, Yu Chen, Yuping Ren, Jiehua Zhou, Junping Ren, Inhan Lee, Xiaoyong Bao

**Affiliations:** 1 Department of Pediatrics, TongJi Hospital, TongJi Medical College, Huazhong University of Science and Technology, Huazhong, China; 2 Department of Pediatrics, University of Texas Medical Branch, Galveston, TX USA; 3 miRcore, Ann Arbor, MI USA; 4 Sealy Center for Environmental Toxicology, University of Texas Medical Branch, Galveston, TX USA; 5 Sealy Center for Molecular Medicine, University of Texas Medical Branch, Galveston, TX USA; 6 Institute for Translational Sciences, University of Texas Medical Branch, Galveston, TX USA; 7 Institute for Human Infections & Immunity, University of Texas Medical Branch, Galveston, TX USA

## Abstract

Chronic exposure to environmental heavy metals is a worldwide health concern. It is acknowledged to be an important cause of lower respiratory tract damage in children. However, the molecular mechanisms underlying the heavy metal-induced cellular stress/toxicity are not completely understood. Small non-coding RNAs (sncRNAs), such as microRNAs (miRNA) and more recently identified tRNA-derived RNA fragments (tRFs), are critical to the posttranscriptional control of genes. We used deep sequencing to investigate whether cellular sncRNA profiles are changed by environmental heavy metals. We found that the treatment of arsenite, an important groundwater heavy metal, leads to abundant production of tRFs, that are ~30 nucleotides (nts) long and most of which correspond to the 5′-end of mature tRNAs. It is unlikely for these tRFs to be random degradation by-products, as the type of induced tRFs is heavy metal-dependent. Three most inducible tRFs and their roles in arsenite-induced cellular responses were then investigated. We identified that p65, an important transcription factor belonging to NF-κB family and also a key factor controlling inflammatory gene expression, is a regulated target of a tRF derived from 5′-end of mature tRNA encoding AlaCGC (tRF5-AlaCGC). tRF5-AlaCGC activates p65, subsequently leading to enhanced secretion of IL-8 in arsenite response. In this study, we also identified that endonuclease Dicer and angiogenin temporally control the induction of tRF5-AlaCGC, providing an insight into the control of tRF biogenesis and subsequently the prevention of cellular damage.

## Introduction

Environmental exposure to arsenite is an ongoing worldwide health problem, with chronic ingestion of contaminated drinking water being the major source of exposure^1^. There are an increasing number of reports showing that chronic exposure to arsenite, including the exposure to drinking-water arsenite, has been correlated with lower respiratory tract damage to cause both malignant and non-malignant lung diseases^2–4^. Despite strong associations between arsenite exposure and respiratory illness, the pathophysiological mechanisms by which arsenite acts on the lung remain largely unknown.

Over the past decade, it has become clear that small non-coding RNAs (sncRNAs) play a significant role in many cellular processes, including cell proliferation^5^, stress responses^6,7^, and host-virus interactions^8,9^. Continuous discoveries on sncRNAs have changed the landscape of human genetics and molecular biology, largely due to the fundamental role of miRNAs-the most extensively studied sncRNA class, as gene regulators^10^. Given their molecular nature and gene regulatory role in broad biological settings, sncRNAs are being recognized as regulatory objectives and innovative intervention tools to control many cellular responses by modifying the expression or gene silencing activity of sncRNAs^11^.

tRNA-derived RNA Fragments (tRFs) are a recently identified class of sncRNAs. Their function and associated molecular mechanism(s) have not been well characterized^12^. There is increasing evidence supporting tRFs functional molecules, but not by-products from random degradation. First, tRFs are produced through specific cleavage of tRNA by endonucleases. Second, tRF expression is regulated by biological events including cellular stresses, cell proliferation, or viral infection. Third, some tRFs have gene trans-silencing activity, control protein translation, and/or carry out biological functions including virus replication control and cell division regulation^13–18^. tRF studies are fairly new and the molecular mechanisms underlying the functions of tRFs are largely unclear.

In this study, we identified that arsenite-treated airway epithelial cells had a significantly higher abundance of tRFs, compared to the cells without the treatment. Three significantly induced tRFs derived from the 5′-end of tRNA-ProTGG (tRF5-ProTGG), tRNA-AlaCGC (tRF5-AlaCGC) and tRNA-GluCTC (tRF5-GluCTC) were selected for studying their roles in arsenite-induced cellular responses. We identified p65, an important transcription factor belonging to NF-κB family and also a key factor controlling inflammatory gene expression, as a regulatory target of tRF5-AlaCGC, but not of the other two tRFs. tRF5-AlaCGC activated p65, subsequently leading to enhanced secretion of an inflammatory cytokine IL-8. We also revealed that the biogenesis of tRF5-AlaCGC was temporarily regulated by endonuclease Dicer and angiogenin (ANG). Taken together with other facts that not all tRNAs were cleaved by heavy metals, and the cleavage of these tRNAs were at the 3′- side of the anticodon loop, we conclude that heavy metal-induced tRFs are functional molecules.

## Materials and Methods

### Cell lines, heavy metals and antibodies

A549 cells (human alveolar type II-like epithelial cells) were from ATCC, Manassas, VA, and maintained as described^13,19,20^. SAE cells (human small airway epithelial cells) were purchased from Lonza (Basel, Switzerland) and maintained as described^21^. Heavy metal compounds sodium arsenite, nickel (II) chloride, and cobalt were purchased from Sigma-Aldrich (Sigma, St. Louis, MO). Primary antibodies against P65 and lamin B were purchased from Millipore (Millipore, Billerica, MA) and Sigma-Aldrich respectively. Primary antibodies against ANG, Dicer and Drosha were from Santa Cruz Biotechnology (Santa Cruz, Dallas, Texas). Horseradish-coupled secondary antibodies were also purchased from Santa Cruz.

### RNA extraction, deep sequencing and RNA mapping

**Figure 1:**
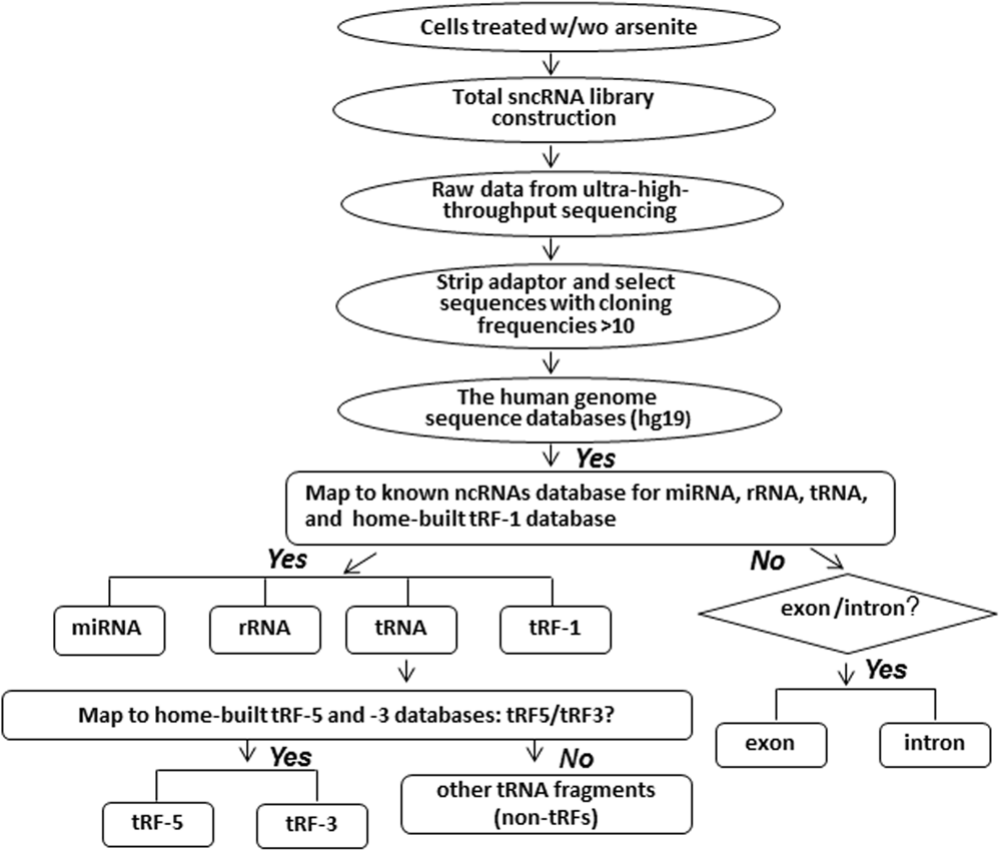
Pipeline of analyses of Illumina high-throughput sequencing data. Flowchart of the sequencing data analyses is depicted.

Total RNAs from A549 cells, treated w/wo heavy metal solution, were extracted by TRIzol Reagents (Thermo Fisher Scientific, Waltham, MA). The RNAs were delivered to the Genomics Core of the University of Texas Medical Branch (UTMB) for small RNAs sequencing. In brief, small RNA libraries were created using the New England Biolabs small RNA library protocol (New England Biolabs). Library construction used a two-step ligation process to create templates compatible with Illumina based next generation sequence (NGS) analysis. Where appropriate, RNA samples were quantified using a Qubit™ fluorometric assay (Thermo Fisher Scientific). RNA quality was assessed using a 2100 Bioanalyzer and the RNA 6000 Pico LabChip® (Agilent Technologies). Library creation uses a sequential addition of first a 3′ adapter sequence followed by a 5′ adapter sequence. A cDNA copy was then synthesized using ProtoScript® II reverse transcriptase (New England Biolabs) and a primer complementary to a segment of the 3′ adapter. Amplification of the template population was performed in 15 cycles (94 °C for 30 sec; 62 °C for 30 sec; 70 °C for 30 sec). The libraries were not size selected. All NGS libraries were indexed. The final concentration of all NGS libraries was determined using a Qubit™ fluorometric assay and the DNA fragment size of each library was assessed using a DNA 1000 high sensitivity chip and an Agilent 2100 Bioanalyzer. Sequence analysis, 2 × 50 bases, was performed on an Illumina Hi-Seq. 1500 using the TruSeq SBS kit v3. The sequence reads ≥ 10 after adaptor sequence removal were used for further classification and small RNAs were mapped using Novoalign software as we previously described in^18,22^ (also shown in Fig. 1). The raw data is deposited to Gene Express Omnibus (https://www.ncbi.nlm.nih.gov/geo/) or available upon request.

### Northern Blot

Northern hybridization for sncRNAs induction was performed, as we previously described^18,22^. Briefly, RNA was separated in 8% or10% denaturing polyacrylamide gel with 7 mol/l urea and then transferred to a positively charged nylon membrane. The membrane was hybridized with ^32^P-labeled probes in ULTRAhyb®-Oligo solution, followed image development.

### Oligo Transfection

Confluent A549 cells were transfected with an antisense oligo (100 nM) against a specific tRF or a control α-oligo using Lipofectamine® 2000 DNA transfection reagent according to manufacturer’s protocol (Invitrogen, Thermo Fisher, Grand Island, NY). At 2 hours (h) post-transfection, cells were treated with/without (w/wo) arsenite (10 μM) for 15 h at 37 °C, followed by sample harvesting.

### Western Blot (WB)

Nuclear extracts of cells were prepared, as we previously described, and quantified with a protein quantification kit from Bio-Rad (Hercules, California). Proteins were fractionated by 10% SDS-PAGE denaturing gels, and transferred to polyvinylidene difluoride membranes, as we previously described^13,23,24^. Membranes were blocked with 5% milk in TBS-Tween 20 and incubated with the proper primary antibody to detect p65. Membranes were then stripped and re-probed for lamin B detection.

### IL-8 Quantification

The level of IL-8 was quantified using human IL-8 Quantikine ELISA Kit from R&D Systems (Minneapolis, MN), according to the manufacturer’s instructions.

### Quantitative Real-time PCR

Total cellular RNAs were extracted using TRIzol reagents (Invitrogen). First strand cDNA was synthesized by using TaqMan RT reagents (ABI, Carlsbad, CA). The RT reaction was performed under the following conditions: 25 °C, 10 minutes; 48 °C, 30 minutes; 95 °C, 5 minutes. Quantitative RT-PCR amplification was performed by using SYBR. Sequence information on green-labeled primers for target genes and internal control 18 S rRNA is available upon request. Quantitative PCR reactions were performed with the FastStart Universal SYBR Green Master (ROX) (Roche, San Francisco, CA) in the ABI 7500 Sequence Detection System using following conditions: initial steps: 50 °C, 2 minutes and 95 °C, 10 minutes; PCR steps: 95 °C, 15 seconds and 60 °C, 1 minute for 40 cycles. The information on RT primers to quantify ANG, Dicer, and Drosha is available upon request.

### Statistical analysis

Data are presented as means ± SEM, and statistically significant differences were assessed by Student’s *t* test for paired or unpaired data, or one-way ANOVA, as appropriate.

## Results

### Heavy metal-induced tRFs

Heavy metal-induced tRFs were first reported for arsenite^25^, a chemical compound which can be inhaled or absorbed through the skin, leading to various types of disease symptoms including death (New Jersey Department of Health and Senior Services. Hazardous Substance Fact Sheet: Sodium Arsenite) &^26^. Although the induction of tRFs was suggested to contribute to arsenite-induced cellular stress, there was no direct evidence. In addition, the study was done in a microarray format, which might leave some tRFs with significance in heavy metal-induced diseases unrevealed. Since the chronic exposure to arsenite has been correlated with lower respiratory tract damage^2–4^, A549 cells, a lower respiratory epithelial cell model, were used and the change of a global sncRNA expression profile was obtained in response to arsenite treatment by Illumina ultra-high-throughput sequencing. These raw data were analyzed as depicted in Fig. 1. In brief, sncRNAs from arsenite-untreated or -treated cells were classified to different groups by mapping them to the miRNA database (miRBase; http://www.mirbase.org), the rRNA database (RDP; http://rdp.cme.msu.edu/), the tRNA database (GtRNAdb; http://gtrnadb.ucsc.edu/), and the Exon-Intron database (EID; http://www.utoledo.edu/med/depts/bioinfo/database.html). In high-throughput sequencing, the cloning frequency provides a digital expression of sncRNAs. Among sncRNAs, no significant changes in miRNAs or rRNA-derived sncRNAs were found in response to arsenite (data not shown). At 6 h post treatment with arsenite, the percentile of tRNA-derived sncRNAs was increased by the fold of 1.6 in A549 cells. The majority of affected tRFs was derived from the 5′-end and for those with induction larger than two by arsenite were listed in Table 1. Since not all the tRNAs were detected to produce tRFs and the sncRNAs were unequally increased in response to the treatment of arsenite, we do not think tRFs as random degraded RNA by-products. More evidence will be provided to demonstrate that sncRNAs from tRNAs are not by-products of random RNA degradation in the manuscript. To investigate whether tRF is also commonly induced by other heavy metals, we treated A549 cells with 5 µM nickel(II) chloride or cobalt(II) chloride, and found that both metals could also induce tRFs (Supplementary Tables I and II). However, the profiles were quite different, suggesting the tRF origins and expression level were heavy metal type dependent, and further supporting that the heavy metal-induced tRFs are not random degraded RNA by-products.

**Table 1:** Summary of tRF-5 series with a relative cloning frequency >0.02‰ (reads per mil) in arsenite-treated samples.

Sequence	Origin	Relative sequencing frequency (‰ of total sequencing reads)	
		Untreated	Arsenite
GGGGGTATAGCTCAGCGGTAGAGCGCGTGCT	AlaTGC	0.13	0.46
GGGGATGTAGCTCAGTGGTAGAGCGCGCTTC	AlaCGC	1.31	7.01
GGGGGTGTAGCTCAGTGGTAGAGCGCGTGCT	AlaAGC	1.00	5.25
GGCTCTGGCGCAATGGATAGCGCATTGGACT	ArgAGA	0.10	11.66
GGGCCAGTGGCGCAATGGATAACGCGTCTGA	ArgACG	0.08	0.83
GACCGCGTGGCCTAATGGATAAGGCGTCTGA	ArgCGG	0.04	6.86
GGCTCCGTGGCGCAATGGATAGCGCATTGGA	ArgTCT	0.28	13.46
GGTCCCATGGTGTAATGGTTAGCACTCTGGA	GlnCAA	0.02	1.59
GGTTCCATGGTGTAATGGTTAGCACTCTGGA	GlnCAG	0.07	49.50
GGTTCCATGGTGTAATGGTAAGCACTCTGGA	GlnCTG	0.06	2.84
GGCCCCATGGTGTAATGGTTAGCACTCTGGA	GlnTTG	0.01	0.72
TCCCTGGTGGTCTAGTGGTTAGGATTCGGCGCT	GluCTC-S	0.01	52.90
TCCCTGGTGGTCTAGTGGTTAGGATTCGGCGCTCAATAATTCTCGGCTGC	GluCTC-L	0.00	33.90
GCATGTGGTTCAGTGGTAGAATTCTCGCCTA	GlyGCC	11.31	23.64
GGTAGCGTGGCCGAGCGGTCTAAGGCGCTGG	LeuAAG	1.322	2.95
AGCAGAGTGGCGCAGCGGAAGCGTGCTGGGC	MetCAT-1	6.494	79.38
AGCAGTGGCGCAGCGGAAGCGTGCTGGGCCC	MetCAT-2	0.13	0.83
GGCTCGTTGGTCTAGGGGTATGATTCTCGCT	ProTGG	2.01	9.01
GGCTTGGTCTAGGGGTATGATTCTCGCTTCA	ProCGG	0.07	8.96
GCTGTGATGGCCGAGTGGTTAAGGCATTGGA	SerCGA	0.03	0.06
GGCCTCGTGGCGCAACGGTAGCGCGTCTGAC	TrpTGG	0.08	1.28
GACCTCGTGGCGCAACGGTAGCGCGTCTGAC	TrpCCA	0.03	4.42
GGCTCCATAGCTCAGGGGTTAGAGCACTGGT	ThrTGT	0.23	1.28

The relative cloning frequency of a tRF was calculated by dividing its read number by the total read number of each experimental group. tRFs were sorted by an alphabetical order of parental tRNA isoforms. For tRNA isoforms sharing the same anticodon, they were sequentially numbered according to the abundance of tRF-5s in the arsenite-treated sample.

### Expression validation of RSV-induced tRFs

**Figure 2:**
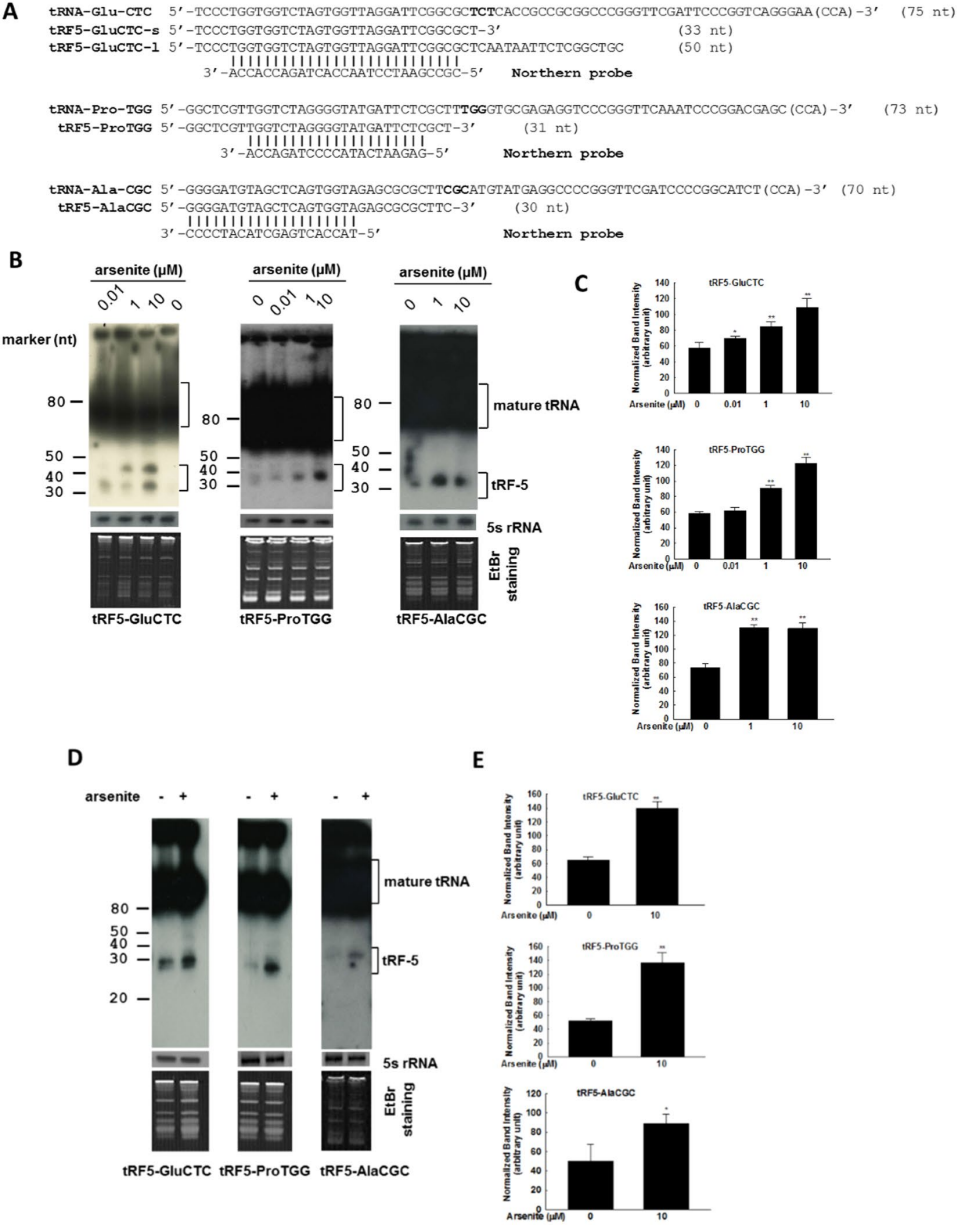
Experimental validation of tRF-5s. (**A**) Sequence alignment of three validated tRF5s with their parental mature tRNAs and Northern probes. The letters in bold indicate the codon sequences. (**B**) Total RNA from A549 cells, treated with arsenite for 6 h at indicated concentrations was loaded to a denaturing polyacrylamide gel for Northern hybridization using probes indicated in panel A. Untreated cells were used as control. Total RNAs stained with ethidium bromide (EtBr) staining and Northern detection on the 5 S rRNA are shown for equal loading. The positions of tRF-5 and mature tRNA are indicated on the right; molecular size markers are indicated on the left. The blot was exposed for 8 h, 1 day, and 5 days for the detection of tRF5-GluCTC, tRF5-ProTGG, and tRF5-AlaCGC, respectively. Data are representative of three independent experiments. (**C**) Densitometric analysis of the tRF bands from three Northern blots was performed for 1B, using the histogram function of Adobe Photoshop (San Jose, CA). Basically, the mean tRF intensity was normalized by the corresponding mean intensity 5 S rRNA and expressed as mean ± standard error (SE). For two bands of tRF5-GluCTC in the same treatment, their mean intensity was first calculated, followed by normalization. (**D,E**) The induction of tRF5-GluCTC, tRF5-ProTGG, and tRF5-AlaCGC were also inducible by arsentite in SAE cells. The Northern blot was done similarly as described in B, while the band intensity was quantified as described in C. Data shown are representative of three independent experiments. * and ** represent *P* < 0.05 and *P* < 0.01 respectively, relative to CN oligo-treated cells.

Here, we selected three tRFs with different fold induction to validate the deep sequencing data, using probes demonstrated in Fig. 2A. The sequencing frequency of tRF5-AlaCGC and tRF5-ProTGG in arsenite-treated cells was in the range of 5–9‰ and their fold induction was more than four. There were two types of arsenite-induced tRF5-GluCTC, with the sequencing frequency much higher than tRF5-AlaCGC and tRF5-ProTGG. Based on their lengths, we named them tRF5-GluCTC-L (long) and tRF5-GluCTC-S (short). The induction of all these tRFs in A549 cells was confirmed by Northern blot (Fig. 2B), demonstrating the fidelity of sequencing data. The normalized band intensity from three experiments were summarized and shown in Fig. 2C. To further confirm the sequencing fidelity, we also used Northern blot to detect tRF5-LysCTT which was not inducible by arsenite according to our sequencing data. As shown in Supplementary Fig. 1, our Northern blot failed to detect tRF5-LysCTT in arsenite-treated sample. We included respiratory syncytial virus (RSV)-infected samples as a positive control to ensure the probing worked properly.

It is interesting that tRF5-GluCTC-S, but not tRF5-GluCTC-L, is also inducible by RSV, a leading cause of the lower respiratory tract infection in the pediatric population^18^, further supporting stimulus-dependent tRF induction.

Although A549 cells are a common cell model for respiratory infection and stress studies^21,27–29^, it is physiologically needed to confirm that primary airway epithelial cells can also produce these tRFs upon the treatment of arsenite. To investigate that, primary cultured human small airway epithelial (SAE) cells were treated with 10 µM arsenite for 15 h, followed by total RNA extraction and Northern blot described in Fig. 2A. We confirmed that tRF5-GluCTC, tRF5-ProTGG, and tRF5-AlaCGC were also inducible by arsenite in SAE cells (Fig. 2D,E), suggesting A549 cells are a feasible cell model to study the cellular response to heavy metal treatment.

### The role of tRFs in arsenite-induced cellular responses

**Figure 3:**
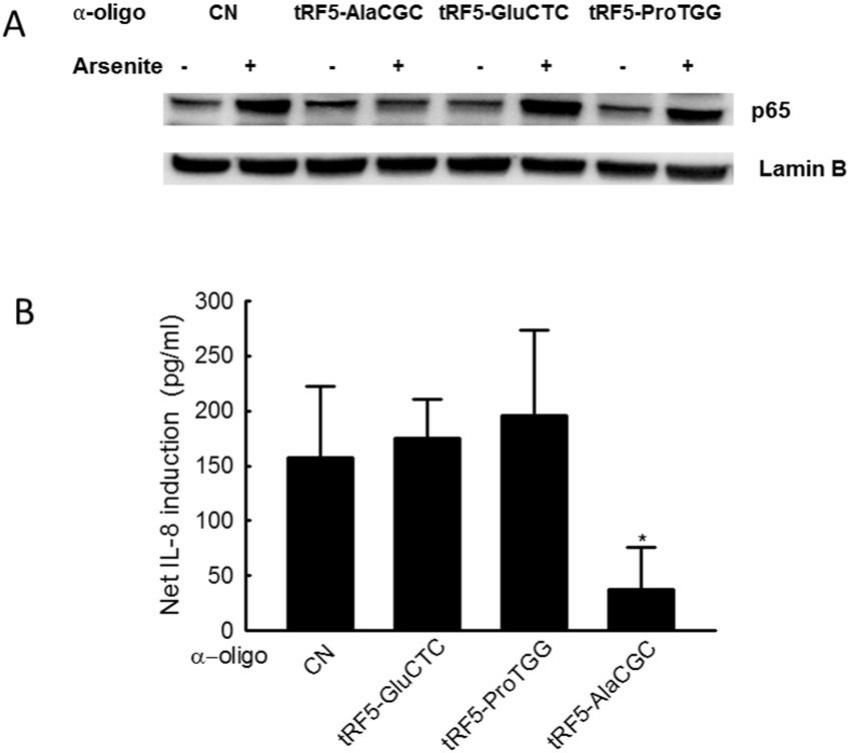
The role of tRFs in arsenite-induced inflammation. (**A**) A549 cells in 6-wells were transfected with 100 nM anti-sense oligos (anti-“tRF5-GluCTC”, -“tRF5-ProTGG”, -“tRF5-AlaCGC” or -“CN”). After 2 h post-transfection, cells were treated with 10 µM arsenite for 15 h. Untreated cells were used as controls. The nuclear fractions were prepared, followed by the SDS-PAGE gel running and Western blot, to detect the nuclear translocation of p65. (**B**) The supernatant of each group was harvested, and its IL-8 was measured by an ELISA kit from R&D (R&D system, Minneapolis, MN). Data shown are representative of three independent experiments. ^*^P < 0.05 relative to CN oligo-treated cells.

Although arsenite is a significant environmental contaminant found in soil and water, and besides its neurobehavioral and carcinogenetic effects, it is also highly associated with lower respiratory tract damage, the molecular mechanism contributing to its impact on cellular responses is largely unknown^2,30,31^. Herein, we investigated whether and how arsenite-induced tRFs contribute to cellular responses to arsenite. Since NF-ĸB, a transcriptional factor, has been shown previously to be responsible for carcinogenesis, apoptosis, and inflammation in arsenite responses^32,33^, we first studied whether tRFs affect the activation of NF-ĸB. As shown in Fig. 3A, airway epithelial cells, transfected with scrambled anti-tRF oligos, had a significant nuclear translocation of p65, a key member of NF-ĸB family, in response to arsenite treatment. Such nuclear translocation was also observed in cells treated with anti-tRF oligos against tRF5-GluCTC (both forms) or tRF5-ProGTT, suggesting an unessential role of these two tRFs in arsenite-induced p65 nuclear translocation. In contrast, cells treated with oligo specifically against tRF5-AlaCGC failed to have arsenite-induced p65 nuclear translocation, demonstrating that tRF5-AlaCGC is a novel regulator of NF-ĸB in arsenite response. Arsenite-induced inflammatory chemokine IL-8 has been previously reported in airway epithelial cells^34^. It is also known that IL-8 is tightly regulated by NF-ĸB in airway epithelial cells^35^. We, therefore, investigated whether arsenite-induced IL-8 can be inhibited by the treatment of anti-tRF5-AlaCGC oligo. As shown in Fig. 3B, there was an IL-8 induction by arsenite, which was significantly decreased by the treatment of anti-tRF5-AlaCGC, further supporting the role of tRF5-AlaCGC in cellular inflammatory response to arsenite.

### Biogenesis of arsenite-induced tRFs

**Figure 4:**
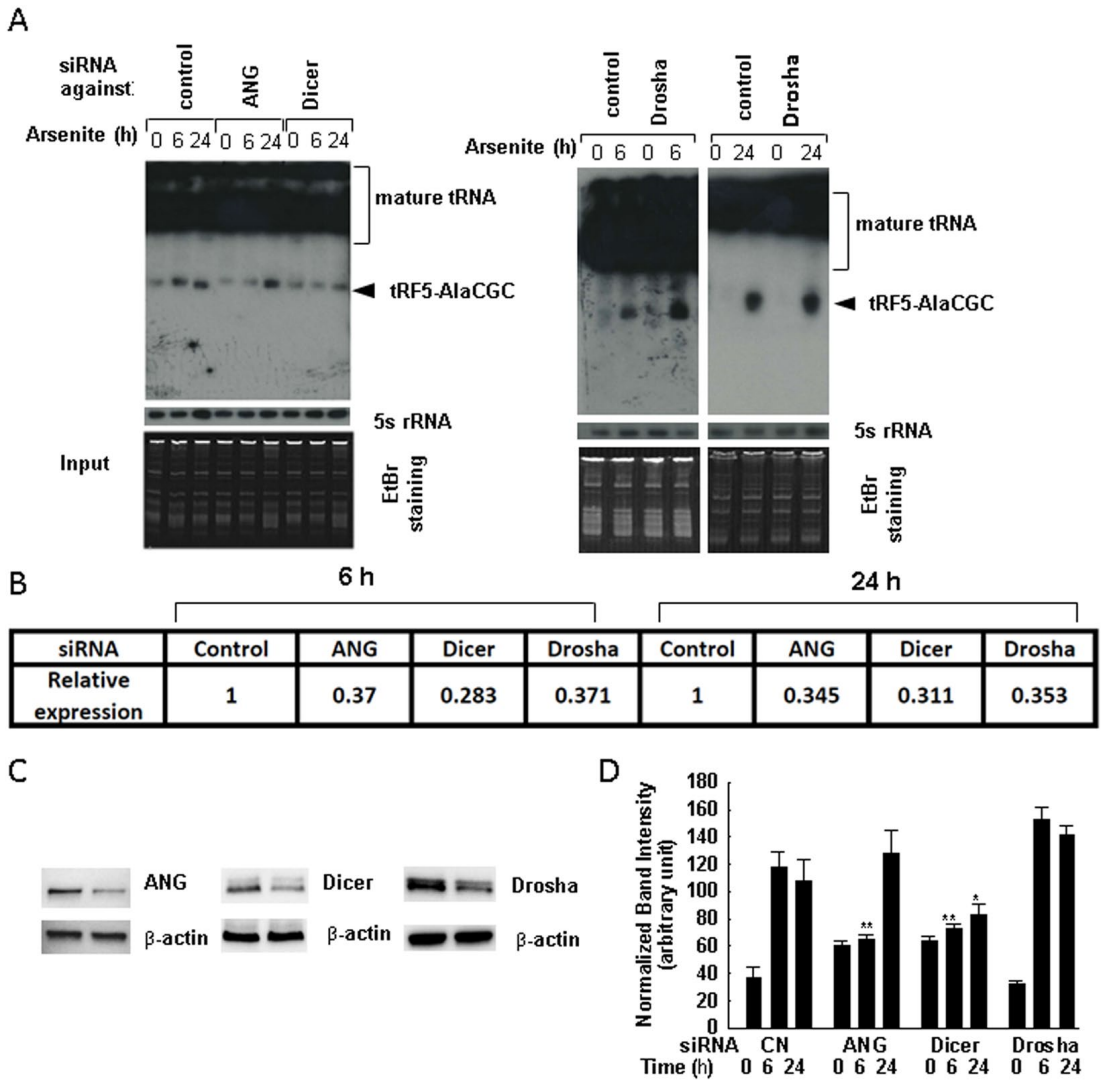
The biogenesis of tRF5-AlaCGC is controlled by angiogenin and Dicer. (**A**) A549 cells were transfected with 100 nM of siRNA against indicated proteins or scrambled siRNA as a negative control. At 40 h post transfection, the cells were treated with arsenite for 6 or 15 h. Cells without the treatment were used as controls. Total RNAs were then subjected to Northern hybridization as described in Fig. 2. 5 S rRNA and EtBr staining were shown for equal loading. (**B**) The suppression of target proteins by each siRNA was confirmed by Real-time PCR. (**C**) The target specific suppression by siRNAs was also confirmed by Western blot after 40 h post transfection. (**D**) Densitometric analysis of the tRF bands from three Northern blots was performed for 4A, similarly as described in Fig. 2C. Data shown are representative of three independent experiments. * and ** represent *P* < 0.05 and *P* < 0.01 respectively, relative to CN oligo-treated cells at corresponding time point of arsenite treatment.

Since not all tRNAs produced tRFs in response to arsenite treatment, we do not think that arsenite-induced tRFs are random degradation products. Close inspection of tRFs in the high-throughput data revealed the 3′-ends of major tRF5 isoforms were located at the anti-codon loop (see Fig. 1A**)**, supporting tRFs are not randomly generated, but produced via a specific pathway. Previously, we and others have shown that both prokaryotes and eukaryotes use ribonucleases to target the tRNA anticodon loop^18,22,36–38^. In particular, a ribonuclease called ANG is responsible for stress- or virus-induced cleavage of tRNA at the anticodon loop to produce tRFs derived from the 5′-end of tRNAs (tRF-5 series)^14,18,22,25^. Dicer, a ribonuclease commonly recognized to be responsible for miRNA biogenesis, was also reported to cleave tRNAs to produce tRFs from the 3′-end of tRNAs (tRF-3 series)^39^, although a recent report demonstrated that some tRF-3 series to be Dicer-independent^40^. Dicer is also responsible for tRNA cleavage on D arms of mature tRNAs in cancer cells to produce tRF5^41^. Whether Dicer contributes to heavy metal-induced tRFs is currently unknown. Since tRF5-AlaCGC is involved in NF-kB-mediated inflammatory response to arsenite, studying the biogenesis mechanism(s) of tRF5-AlaCGC may provide an insight into arsenite-induced inflammation control. We, therefore, suppressed several types of ribonucleases, including ANG and those responsible for miRNA production, by their respective siRNA, and measured tRF5-AlaCGC in arsenite-treated A549 cells. As shown Fig. 4, when ANG or Dicer was suppressed by the siRNA, the induction of tRF5-AlaCGC by arsenite at early time post-treatment was significantly decreased (>50%). The importance of ANG seemed faded along the treatment, while Dicer continued to be critical in producing tRF5-AlaCGC. Interestingly, Drosha knockdown, another ribonuclease responsible for producing pri-miRNA in the nuclear compartment, did not affect the induction of tRF5-AlaCGC, demonstrating different pathways for the induction of tRFs and miRNAs. This also suggested that the tRF cleavage did not occur in the nuclear compartment, which is consistent to cytosol presence of tRFs in the cells^18,22^. Although ANG has been demonstrated in the previous study in mediating arsenite-induced tRFs, their role was focused on the early time point post treatment^14^. This is the first report demonstrating a temporal role of ANG in tRF biogenesis in the context of heavy metal treatment. We also discovered that Dicer can produce tRF-5 series by cleaving tRNA at the anti-codon loop, in addition to its previously described role in generating tRF-3 series and tRF-5 series ending at D arms of mature tRNAs^39,41^. Collectively, our data demonstrated that ANG and Dicer play a role in inducing tRF5-AlaCGC by arsenite. However, ANG only contributes to its early induction.

## Discussion

In this study, we demonstrated that tRF induction is a common biological event in response to heavy metal exposures. Depending upon the type of heavy metal, the profile of induced tRFs varied. However, induced tRFs were always derived from a limited population of mature tRNAs, regardless of which heavy metal was used for the induction. Structurally, the 3′-ends of induced tRFs ended nearly by the anticodon loop. More importantly, a tRF from arsenite-treated cells was proved to be functional in arsenite-induced p65 activation and IL-8 production. All of these supported that tRFs induced by heavy metals were not random degraded byproducts, but functional molecules with biological importance. This discovery may launch a new angle to study the cellular/stress responses to the exposures of heavy metals.

The induction of tRFs is more or less mediated by ribonucleases. As mentioned, Dicer is responsible for the biogenesis of some tRF3s and D-arm cleaved tRF5s^39,41^. In sex hormone-dependent cancers, ANG seems important to generate tRF5 via targeting the anticodon loop^42^. In response to RSV infection, we found that ANG is responsible for tRNA cleavage on the anticodon loop^18,22^. In prostate cancer, ELAC2 is responsible for generating tRFs derived from 3′ end of tRNA precursor transcript^43^. Overall, these studies seemed to support specific cleavage of tRNA by a specific nuclease. Interestingly, our study revealed that both ANG and Dicer are responsible for generating a tRF5 with its 3′-end in the anticodon loop. Why cells need two enzymes to temporally regulate the tRF biogenesis in response to arsenite needs to be examined further. It is well known that Drosha is in the nuclear compartment and also a key enzyme to cleave a long RNA primary transcript known as a pri-miRNA to produce a stem-loop structure of about 70 base pairs long, known as a pre-miRNA. Cytosolic Dicer then cleaves pre-miRNAs to produce miRNAs^44^. Therefore, our study clearly demonstrated that the biogenesis of tRF is different from that of miRNA, as Drosha was not involved in the tRF biogenesis. The independence of tRF on Drosha also suggested that tRF cleavage did not occur in the nuclear compartment, which is consistent to what we reported for tRFs’ cytosolic presence^18,22^. Other than the mechanism by which ribonucleases use to generate specific tRFs in certain biological settings, it is also possible that the enzymes actually cleave all tRNAs. However, some tRFs are not detectable due to their instability. Currently we do not know whether some tRFs are regulated at the post-cleavage steps. However, for tRF5-AlaCGC, it is inducible by arsenite but not by cobalt. Since both treatments were done in A549 cells, it is less likely for tRF5-AlaCGC to be induced by cobalt but unstable in A549 cells. As discussed, arsenite is an important health risk factor and its contribution to respiratory illness is significant, with mechanisms largely unknown^45^. The investigated response to arsenite treatment is mainly oxidative stress and its regulation at the protein level^46,47^. It has been suggested that cells deficient in eIF2α phosphorylation have a higher accumulation of tRNA fragments derived from initiator-tRNA^Met^ in response to arsenite, which may affect the rates of protein synthesis^16^. The accumulation of tRF5-MetCTA by arsenite was also observed in our study (Table 1). tRFs were also suggested to be involved in granule formation via binding to translational silencer YB-1^48^. In this study, we investigated whether there are any other mechanisms than tRF-controlled protein synthesis contributing to heavy metal-induced pathogenesis, we discovered that a tRF actually was involved in the regulation of the activation of transcription factor p65 and subsequently enhanced the IL-8 secretion in response to arsenite treatment. In the future, we will investigate the molecular mechanism underlying the interaction of tRF5-AlaCGC and p65. Overall, this study generated a new study angle to use tRF to examine mechanisms underlying pathogenic diseases by heavy metals, including arsenite, cobalt and nickel^49–52^.

## Electronic supplementary material

Supplementary figure and tables
