# Supplementary material for: A tRNA-derived RNA Fragment Plays an Important Role in the Mechanism of Arsenite -induced Cellular Responses
Source: Sci Rep. 2018 Nov 15;8:16838. doi: 10.1038/s41598-018-34899-2 (PMC6237853; doi:10.1038/s41598-018-34899-2)
Supplement: Supplementary file 1 — Supplementary figure and tables [file 41598_2018_34899_MOESM1_ESM.pdf]

A tRNA-derived RNA Fragment Plays an Important Role in the Mechanism of Arsenite -induced Cellular Responses

Shengxuan Liu, Yu Chen, Yuping Ren, Jiehua Zhou, Junping Ren, Inhan Lee,& Xiaoyong Bao

```
tRNA-Lys-CTT 5'-GCCCGGCTAGCTCAGTCGGTAGAGCATGAGACTCTTAATCTCAGGGTCGTGGGTTCGAGCCCCACGTTGGGCG(CCA)-3'(73 nt)
tRF5-LysCTT  5'-GCCCGGCTAGCTCAGTCGGTAGAGCATGAG-3'       (30 nt, 14-fold induction)
                    ||||||||||||||||||||||||
                3'-CCGATCGAGTCAGCCATCTCGTAC-5'      Northern probe
```

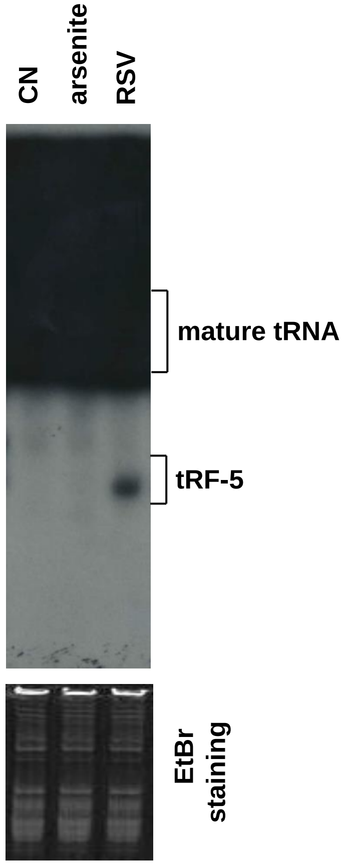

tRF5-LysCTT

		Relative sequencing frequency (‰ of total sequencing reads)	
**Sequence**	**Origin**	**Untreated**	**NiCl**
GGGGGTATAGCTCAGCGGTAGAGCGCGTGCT	AlaAGC	0.01	0.05
GGGGATGTAGCTCAGTGGTAGAGCGCGCTTC	AlaCGC	0.13	0.39
GGGGGTGTAGCTCAGTGGTAGAGCGCGTGCT	AlaTGC	0.10	0.43
GACCCAGTGGCCTAATGGATAAGGCATCAGC	ArgCCG	0.05	0.21
GACCGCGTGGCCTAATGGATAAGGCGTCTGA	ArgTCG	0.00	0.02
GGCTCCGTGGCGCAATGGATAGCGCATTGGA	ArgTCT-A	0.03	0.17
GGCTCTGGCGCAATGGATAGCGCATTGGACT	ArgTCT-B	0.01	0.06
GCATTGTGGTTCAGTGGTAGAATTCTCGCCT	GlyGCC -A	40.81	86.54
GCATGTGGTTCAGTGGTAGAATTCTCGCCTA	GlyGCC -B	1.13	3.32
GCGCCGCTGGTGTAGTGGTATCATGCAAGAT	GlyGCC-C	0.52	1.49
GGTCCCATGGTGTAATGGTTAGCACTCTGGA	GlnCAA	0.00	0.02
GGTTCCATGGTGTAATGGTTAGCACTCTGGA	GlnCAG	0.07	0.44
GGTTCCATGGTGTAATGGTAAGCACTCTGGA	GlnCTG	0.06	0.03
GGCCCCATGGTGTAATGGTTAGCACTCTGGA	GlnTTG	0.00	0.01
GCCCGGCTAGCTCAGTCGGTAGAGCATGAGA	LysCTT-A	76.70	241.53
GCCCGGCTAGCTCAGTCGGTAGAGCATGGGA	LysCTT-B	22.33	47.80
GCCCGGATAGCTCAGTCGGTAGAGCATCAGA	LysTTT-C	1.74	5.92
GGCCGGTTAGCTCAGTTGGTTAGAGCGTGGT	LleAAT	0.02	0.09
AGCAGAGTGGCGCAGCGGAAGCGTGCTGGGC	MetCAT-A	0.65	4.07
AGCAGTGGCGCAGCGGAAGCGTGCTGGGCCC	MetCAT-B	0.01	0.12
GCCTCGTTAGCGCAGTAGGTAGCGCGTCAGT	MetCAT-C	0.01	0.04
GGCTTGGTCTAGGGGTATGATTCTCGCTTCA	ProTGG	0.20	1.51
GACGAGGTGGCCGAGTGGTTAAGGCGATGGA	SerGCT	0.29	0.72
GGCTCCATAGCTCAGGGGTTAGAGCACTGGT	ThrTGT	0.02	0.05

Supplementary Table I: Summary of tRF-5 series with a relative cloning frequency > 0.02‰ (reads per mil) in samples treated with nickel for 6 h

		Relative sequencing frequency (‰ of total sequencing	
**Sequence**	**Origin**	**Untreated**	**Cobalt**
GGGGGTATAGCTCAGCGGTAGAGCGCGTGCT	AlaAGC	0.01	0.03
GGCTCCGTGGCGCAATGGATAGCGCATTGGA	ArgTCT	0.03	0.08
GCATTGTGGTTCAGTGGTAGAATTCTCGCCT	GlyGCC 1	40.81	90.65
GCATGTGGTTCAGTGGTAGAATTCTCGCCTA	GlyGCC 2	1.13	3.00
GGTTCCATGGTGTAATGGTTAGCACTCTGGA	GlnCAG	0.07	0.22
GGTTCCATGGTGTAATGGTAAGCACTCTGGA	GlnCTG	0.01	0.02
GGCCGGTTAGCTCAGTTGGTTAGAGCGTGGT	LleAAT	0.02	0.08
GGCTTGGTCTAGGGGTATGATTCTCGCTTCA	ProCGG	0.01	0.02
GACGAGGTGGCCGAGTGGTTAAGGCGATGGA	SerGCT	0.29	1.04
GTCACGGTGGCCGAGTGGTTAAA	SerCGA	0.02	0.08

Supplementary Table II,Summary of tRF-5 series with a relative cloning frequency > 0.02‰ (reads per mil) in cobalt-treated samples

Supplementary Figure Legends

S. Figure 1. Total RNAs from A549 cells, treated with arsenite (lane 2) as described in Figure 2 or infected with RSV (lane 3, a positive control) at the multiplicity of infection of 3 for 6 h, were loaded to a denaturing polyacrylamide gel for Northern hybridization using a probe indicated in S. Figure 1 A. Untreated cells were used as control. Total RNAs stained with ethidium bromide (EtBr) staining are shown for equal loading. The positions of tRF5-LycCTT and its corresponding mature tRNA are indicated on the right.

S. Table I. Summary of tRF-5 series with a relative cloning frequency > 0.02‰ (reads per mil) in NiCl-treated samples. The relative cloning frequency of a tRF was calculated and sorted as described in Table I. For tRNA isoforms sharing the same anticodon, they were in alpha-beta order according to the abundance of tRF-5 series in the NiCl-treated sample.

S. Table II. Summary of tRF-5 series with a relative cloning frequency > 0.02‰ (reads per mil) in cobalt-treated samples. The relative cloning frequency of a tRF was calculated and sorted as described in Table I. For tRNA isoforms sharing the same anticodon, they were sequentially numbered according to the abundance of tRF5s in the cobalt-treated sample.
